# miR-9-5p and miR-221-3p Promote Human Mesenchymal Stem Cells to Alleviate Carbon Tetrachloride-Induced Liver Injury by Enhancing Human Mesenchymal Stem Cell Engraftment and Inhibiting Hepatic Stellate Cell Activation

**DOI:** 10.3390/ijms25137235

**Published:** 2024-06-30

**Authors:** Lihong He, Jianwei Xu, Ping Huang, Yu Bai, Huanhuan Chen, Xiaojing Xu, Ya’nan Hu, Jinming Liu, Huanxiang Zhang

**Affiliations:** Department of Cell Biology, MOE Key Laboratory of Geriatric Diseases and Immunology, Suzhou Medical College of Soochow University, Suzhou 215123, China; helh@suda.edu.cn (L.H.); xujianwei@gmc.edu.cn (J.X.);

**Keywords:** human umbilical cord-derived MSCs, homing, cell engraftment, miR-9-5p, miR-221-3p, liver injury

## Abstract

Mesenchymal stem cells (MSCs) have shown great potential for the treatment of liver injuries, and the therapeutic efficacy greatly depends on their homing to the site of injury. In the present study, we detected significant upregulation of hepatocyte growth factor (HGF) in the serum and liver in mice with acute or chronic liver injury. In vitro study revealed that upregulation of miR-9-5p or miR-221-3p promoted the migration of human MSCs (hMSCs) toward HGF. Moreover, overexpression of miR-9-5p or miR-221-3p promoted hMSC homing to the injured liver and resulted in significantly higher engraftment upon peripheral infusion. hMSCs reduced hepatic necrosis and inflammatory infiltration but showed little effect on extracellular matrix (ECM) deposition. By contrast, hMSCs overexpressing miR-9-5p or miR-221-3p resulted in not only less centrilobular necrosis and venous congestion but also a significant reduction of ECM deposition, leading to obvious improvement of hepatocyte morphology and alleviation of fibrosis around central vein and portal triads. Further studies showed that hMSCs inhibited the activation of hepatic stellate cells (HSCs) but could not decrease the expression of TIMP-1 upon acute injury and the expression of MCP-1 and TIMP-1 upon chronic injury, while hMSCs overexpressing miR-9-5p or miR-221-3p led to further inactivation of HSCs and downregulation of all three fibrogenic and proinflammatory factors TGF-β, MCP-1, and TIMP-1 upon both acute and chronic injuries. Overexpression of miR-9-5p or miR-221-3p significantly downregulated the expression of α-SMA and Col-1α1 in activated human hepatic stellate cell line LX-2, suggesting that miR-9-5p and miR-221-3p may partially contribute to the alleviation of liver injury by preventing HSC activation and collagen expression, shedding light on improving the therapeutic efficacy of hMSCs via microRNA modification.

## 1. Introduction

As multipotent adult stem cells that can be easily isolated and proliferated in vitro and possess strong plasticity and immunomodulatory function but no immunogenicity, mesenchymal stem cells (MSCs) are emerging as the most ideal source for allogeneic transplantation. MSCs derived from neonatal human umbilical cords (hMSCs) are more primitive than those from post-natal tissues and therefore, have been extensively studied for potential clinical application [1,2,3,4,5].

MSCs have shown great promise for the treatment of liver injuries, and multiple mechanisms are involved, including differentiation into functional hepatocyte-like cells and secretion of paracrine factors to promote liver regeneration, immunomodulation, and suppression of inflammatory infiltration, degradation of extracellular matrix (ECM), and alleviation of fibrosis, emerging as the most appealing regenerative medicine for the treatment of advanced cirrhosis and liver failure instead of/before whole organ transplantation [6,7,8]. The therapeutic efficacy greatly depends on their migration toward injury and the resultant engraftment efficiency, as MSCs are commonly infused by intravenous injection or direct injection into the hepatic parenchyma and thus must migrate a long way with bloodstream or within the liver to reach the sites of injury [6,8]. Growth factors and cytokines released from tissues around injury are reported to regulate MSC migration and tropism to target sites [6,9,10,11,12,13]. In rodents and patients with liver steatosis, fibrosis, cirrhosis, or carcinoma, hepatocyte growth factor (HGF), which has been shown to induce MSC migration in vitro [14,15], is excessively upregulated [16,17,18,19,20] and thus may contribute to recruiting MSCs to the injured liver [12]. Pre-treatment of MSCs with HGF or overexpression of c-Met in MSCs improves their repair of liver injury by increasing their homing [21,22,23]. Therefore, strategies that can increase MSC migration toward HGF may enhance their homing and engraftment, leading to improvement of therapeutic efficacy.

miRNAs, the small noncoding RNAs of 18–25 nucleotides, play important roles in regulating MSC migration [24]. We have previously reported that overexpression of miR-9-5p or miR-221-3p promoted the migration of rat bone marrow-derived MSCs toward HGF [25,26]. In the present study, in mice with carbon tetrachloride (CCl_4_)-induced acute or chronic liver injury, we detected significant upregulation of HGF in the serum and liver. In vitro study revealed significant promotion of hMSC migration toward HGF by miR-9-5p or miR-221-3p as with previous reports of rat MSCs. These findings let us speculate that miR-9-5p and miR-221-3p may promote hMSCs’ homing to the injured liver and increase their engraftment, thereby improving therapeutic efficiency. In the present study, we investigated the engraftment and therapeutic outcomes of hMSCs overexpressing miR-9-5p or miR-221-3p for the treatment of liver injuries and tried to explore the underlying cellular and molecular mechanisms.

## 2. Results

### 2.1. miR-9-5p and miR-221-3p Promote the Migration of hMSCs in Response to HGF

hMSCs derived from Wharton’s jelly of umbilical cord exhibited a typical fibroblast-like morphology after three passages of culture, expressing the MSC markers CD44, CD90, CD105, and CD73, but not the hematopoietic markers CD34 and CD45 [9,27], and could differentiate into osteoblasts, chondrocytes, and adipocytes upon appropriate osteogenic, chondrogenic, and adipogenic induction (Supplementary Figure S1).

**Figure 1:**
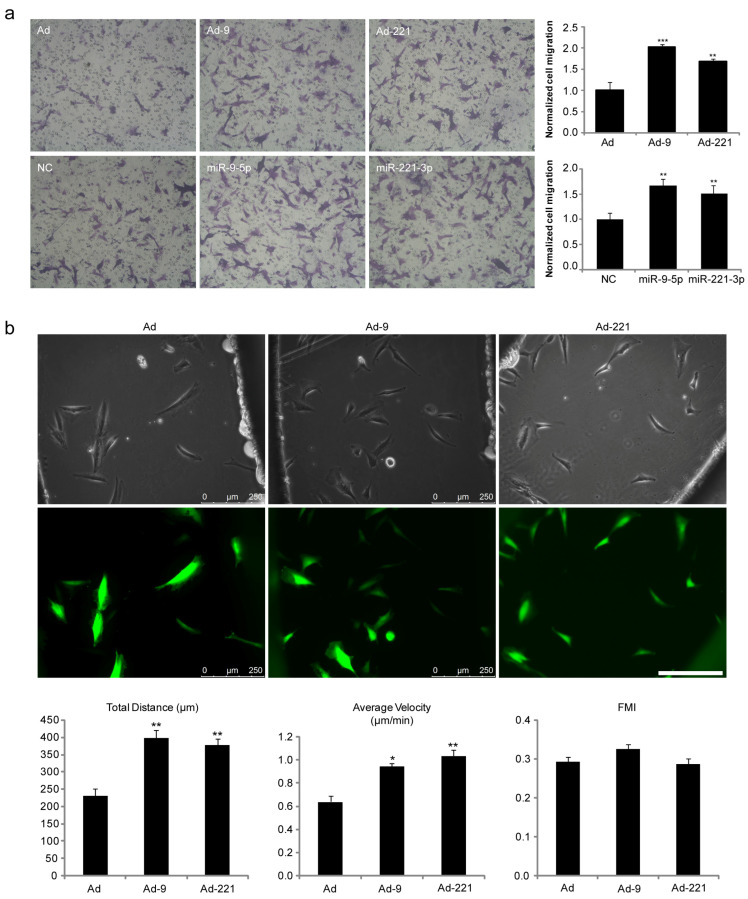
miR-9-5p and miR-221-3p promote the migration of hMSCs toward HGF in vitro. After infection with Ad, Ad-9, or Ad-221 or transfection with negative control (NC), miR-9-5p mimic, or miR-221-3p mimic, hMSCs were tested for migration. (**a**) Transfilter migration of hMSCs toward HGF using a Boyden chamber. HGF (50 ng/mL) was added to the lower compartment of the Boyden chamber. Cells were allowed to migrate toward HGF for 6 h at 37 °C in a 5% CO_2_ humidified incubator. Left panel: Representative images of migratory cells/field on the membrane underside, scale bar = 250 μm. Right panel: Quantitative results of cell transfilter migration induced by HGF. Data are shown as mean ± SEM from three independent experiments (** *p* < 0.01, *** *p* < 0.001, compared with Ad or NC). (**b**) miR-9-5p and miR-221-3p promoted motility but not migration persistence of hMSCs in response to an HGF gradient using a Dunn chamber. Upper panel: hMSCs over the annular bridge between the inner and outer wells of the chamber observed under phase-contrast and fluorescence optics. Cell migration was recorded continuously by time-lapse frame grabbing for 6 h in humidified 5% CO_2_ at 37 °C. Scale bar = 250 μm. Lower panel: Quantitative results of total distance (μm), migration velocity (μm/min), and the forward migration index (FMI). Data are shown as mean ± SEM from three independent experiments (* *p* < 0.05, ** *p* < 0.01, compared with Ad).

To upregulate the expression of miR-9-5p or miR-221-3p and label cells, we infected hMSCs with recombinant adenovirus (Ad) carrying the sequence for the green fluorescent protein (GFP) and the precursor sequence for miR-9-5p or miR-221-3p (Ad-9, Ad-221) or transfected with miR-9-5p mimic or miR-221-3p mimic for 48 h and then incubated with Hoechst 33342 (H33342, Supplementary Figure S2a,b). The HGF-induced transfilter migration of these cells was evaluated using a microchemotaxis Boyden chamber. The results showed that overexpression of miR-9-5p or miR-221-3p elevated hMSC migration by about 2.0-fold and 1.7-fold, respectively (Figure 1a). No effects on hMSC proliferation or apoptosis were observed (Supplementary Figure S3).

Next, we visualized the migratory behavior in response to HGF using a Dunn chamber under time-lapse video microscopy (Figure 1b, Supplementary Videos S1–S3). As shown in Figure 1b, the average distance and the average migration speed were significantly higher in hMSCs overexpressing miR-9-5p or miR-221-3p, while the forward migration index (FMI), which reflects the directional persistence of cell migration, exhibited little variance, suggesting that miR-9-5p and miR-221-3p promote the motility but not the directional persistence of hMSCs.

### 2.2. miR-9-5p and miR-221-3p Promote hMSC Homing to Liver Injury and Improve Macroscopic Liver Appearance

There is evidence showing that the expression of HGF was upregulated upon liver injury [16,19,20]. In this study, we detected 3.6-fold and 4.8-fold upregulation of hepatic HGF transcripts and higher serum HGF concentrations of about 22 ng/mL and 24 ng/mL in mice with acute or chronic liver injury compared to normal mice (0.13 ng/mL, Supplementary Figure S4).

**Figure 2:**
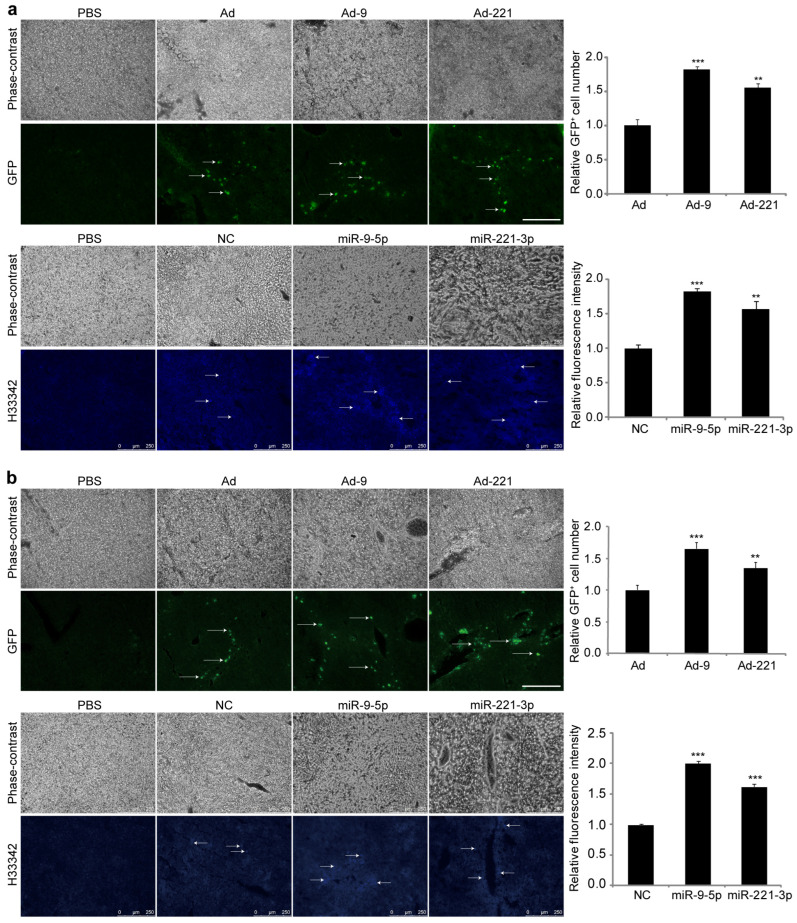
Engraftment of hMSCs after infusion in mice with acute or chronic liver injuries for 7 d. hMSCs that were infected with Ad, Ad-9, or Ad-221, or transfected with NC, miR-9-5p mimic, or miR-221-3p mimic and labeled with H33342 were transplanted to mice with acute (**a**) or chronic (**b**) liver injuries for 7 d (*n* = 5 per group). Representative images (scale bar = 250 μm) with GFP-positive or H33342-labeled transplanted cells are shown. Arrows show the engraftment of transplanted cells. GFP^+^ cell number and fluorescence intensity of H33342 were quantified using ImageJ software 1.54. Data are shown as mean ± SEM from at least three independent experiments (** *p* < 0.01, *** *p* < 0.001, compared with Ad or NC).

After peripheral infusion for 7 days, hMSCs could be observed in both the acutely and chronically injured livers, as detected by GFP and blue fluorescence in cryosections (Figure 2). Higher intensity and wider distribution of fluorescence were found in livers receiving hMSCs overexpressing miR-9-5p or miR-221-3p, mostly localized around central veins and portal triads (Figure 2). These results demonstrate that miR-9-5p and miR-221-3p promote hMSC homing to the injured liver and enhance their engraftment.

Livers were found to enlarge in size and increase in weight after acute or chronic injury, leading to a higher liver/body weight ratio; infusion of hMSCs overexpressing miR-9-5p or miR-221-3p significantly decreased the liver/body weight ratio, while little effect was observed in hMSCs (Supplementary Figure S5a,b). In comparison with hMSCs, miR-9-5p- or miR-221-3p-modification led to a more obvious improvement in the appearance and texture of livers with chronic injury, which exhibited a reddish-brown color, soft texture, and smooth surface with significantly fewer nodules, while livers with chronic injury exhibited a dull color and hard texture with a lot of white or yellow nodules on the surface 

Taken together, peripheral infusion of hMSCs overexpressing miR-9-5p or miR-221-3p in mice with acute or chronic liver injury results in higher engraftment, lower liver/body weight ratio, and more obvious improvement of the appearance and texture of chronically injured liver, indicating that miR-9-5p and miR-221-3p may improve the therapeutic efficacy of hMSCs by promoting their homing and engraftment.

### 2.3. hMSCs Overexpressing miR-9-5p or miR-221-3p Ameliorate CCl_4_-Induced Microscopic Hepatic Injury

**Figure 3:**
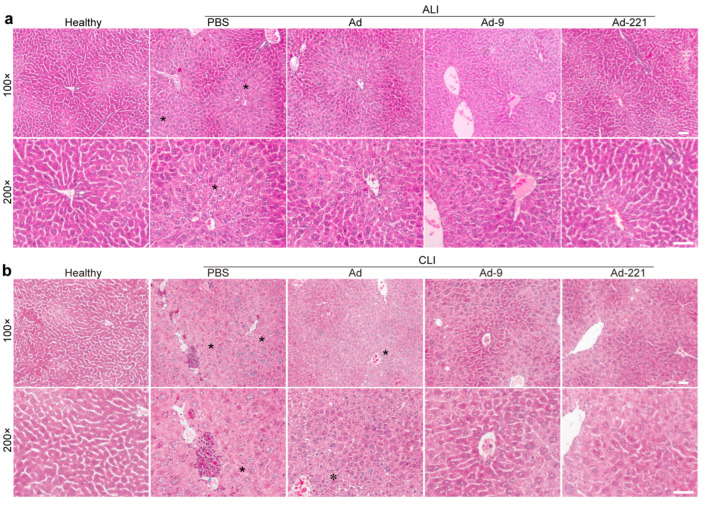
hMSCs overexpressing miR-9-5p or miR-221-3p improved hepatic microscopic architecture. Healthy mice or mice with CCl_4_-induced acute (**a**) and chronic (**b**) liver injury that received PBS or hMSCs for 7 d were cryosectioned and stained with hematoxylin and eosin (*n* = 5 per group). Asterisks show zonal centrilobular hepatocyte necrosis. Scale bar = 100 μm.

Further, we performed histochemical analysis of liver sections to examine the cellular and histochemical changes after cell infusion for 7 d. Acute injury resulted in significant centrilobular necrosis and venous congestion; upon chronic injury, more severe hepatocyte necrosis and inflammatory cell congestion and infiltration were observed, indicating extensive damage to lobular architecture (Figure 3). Transplantation of hMSCs reduced centrilobular necrosis and venous congestion, while overexpression of miR-9-5p or miR-221-3p resulted in further alleviation of these damages (Figure 3), especially in animals with chronic injuries, leading to more obvious improvement in hepatocyte morphology around central vein and portal triads than in unmodified hMSCs (Figure 3b).

**Figure 4:**
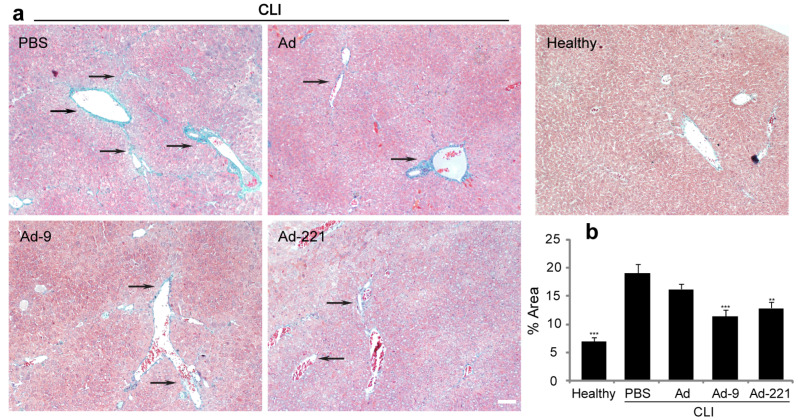
hMSCs overexpressing miR-9-5p or miR-221-3p reduced ECM deposition after peripheral infusion for 7 d. Healthy mice and mice with chronic liver injury received PBS or hMSCs for 7 d (*n* = 5 per group). (**a**) Representative images of ECM deposition in different groups using Masson’s trichrome staining (scale bar = 100 μm). Arrows indicate the accumulation of ECM. (**b**) Quantification of ECM area using ImageJ software 1.54. Data are shown as mean ± SEM of at least three independent experiments (** *p* < 0.01, *** *p* < 0.001, compared with PBS).

In addition to severe hepatocyte necrosis and inflammation, chronic injury also included excessive accumulation of ECM (Figure 4), leading to fibrosis. Infusion of hMSCs resulted in little reduction in ECM, and ECM deposition was still obvious around portal triads and within lobules (Figure 4). By contrast, hMSCs overexpressing miR-9-5p or miR-221-3p significantly decreased ECM deposition (Figure 4a), and quantification of the relative ECM area clearly demonstrated the reduction (Figure 4b), suggesting the alleviation of fibrosis.

### 2.4. hMSCs Overexpressing miR-9-5p or miR-221-3p Alleviate Liver Injuries through Inactivation of HSCs

**Figure 5:**
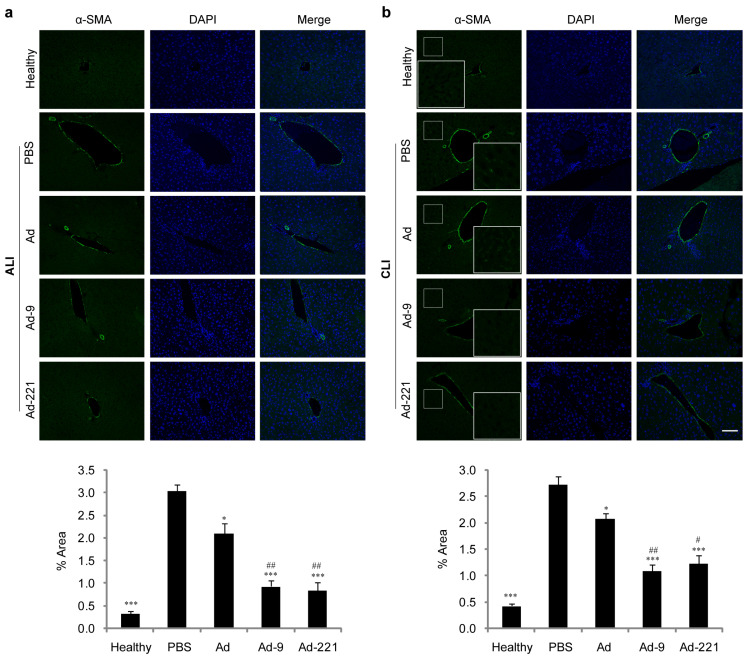
hMSCs overexpressing miR-9-5p or miR-221-3p suppress HSC activation after peripheral infusion for 7 d. Shown are representative images of α-SMA-positive cells (green, scale bar = 100 μm) based on immunofluorescence staining of α-SMA in acutely (**a**) and chronically (**b**) injured livers (*n* = 5 per group). High magnification views in insets show the expression of α-SMA in the perisinusoidal space of hepatic parenchyma. α-SMA expression was quantified using ImageJ software 1.54. Data are shown as mean ± SEM from at least three independent experiments (* *p* < 0.05, *** *p* < 0.001, compared with PBS; # *p* < 0.05, ## *p* < 0.01, compared with Ad).

Upon injury, hepatic stellate cells (HSCs), the resident mesenchymal cells located in the space of Disse in a quiescent state in an intact liver, transdifferentiate into an activated myofibroblast-like phenotype and produce ECM to initiate a tissue repair mechanism, but eventually lead to fibrosis or even cirrhosis in case of persistent injury [28,29,30]. As the definitive marker of activated HSCs, α-smooth muscle actin (α-SMA) was upregulated in the vascular walls around the central vein and portal tracts upon acute injury but decreased after infusion of hMSCs for 7 d; a further decrease occurred upon infusion of hMSCs overexpressing miR-9-5p or miR-221-3p (Figure 5a), suggesting less activation of HSCs.

Upon chronic injury, α-SMA was upregulated not only in the vascular walls around the central vein and portal tracts but also within lobules in the perisinusoidal space of hepatic parenchyma (Figure 5b). Infusion of hMSCs decreased the expression of α-SMA, while overexpression of miR-9-5p or miR-221-3p resulted in a further decrease, nearly undetectable in the perisinusoidal space of parenchyma (Figure 5b), indicating a remarkable inhibition of HSC activation by miR-9-5p- or miR-221-3p-modified hMSCs.

**Figure 6:**
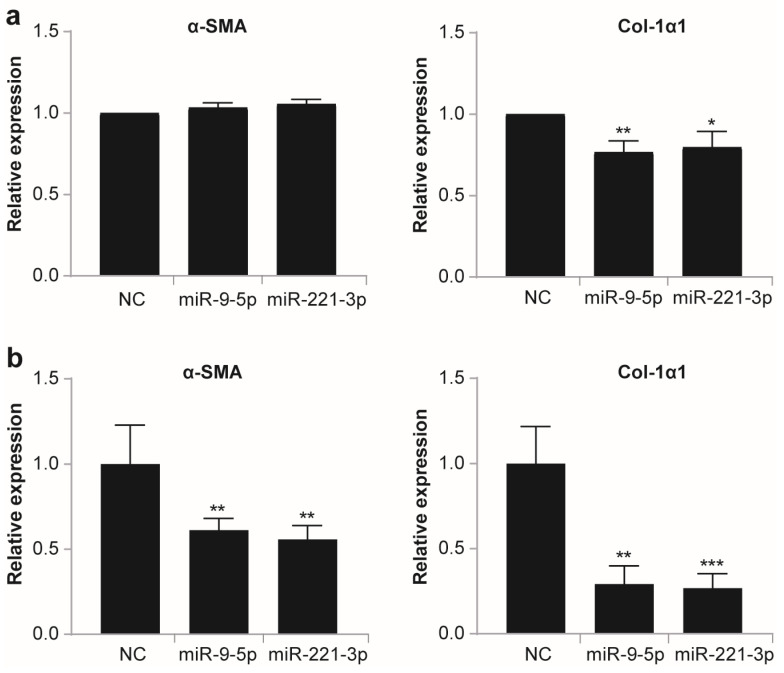
Effects of hMSC-conditioned medium and overexpression of miR-9-5p or miR-221-3p on HSC activation and procollagen expression. Activated human hepatic stellate cell line LX-2 at 60–70% confluence was incubated in the conditioned medium of hMSCs overexpressing miR-9-5p or miR-221-3p (**a**) or directly transfected with miR-9-5p mimic or miR-221-3p mimic for 48 h (**b**), and then the mRNA level of α-SMA and Col-1α1 were quantified using qRT-PCR. Data are shown as mean ± SEM from at least three independent experiments (* *p* < 0.05, ** *p* < 0.01, *** *p* < 0.001, compared with NC).

In vitro treatment of LX-2, an activated human hepatic stellate cell line, with conditioned medium (CM) of hMSCs overexpressing miR-9-5p or miR-221-3p significantly decreased the mRNA level of collagen-1α1 (Col-1α1) in LX-2 but showed little effect on the expression of α-SMA (Figure 6a), suggesting that paracrine function of hMSCs overexpressing miR-9-5p or miR-221-3p may mediate the alleviation of liver fibrosis by decreasing the expression of procollagen in activated HSCs, though current CM treatment is insufficient to reverse the activated LX-2 phenotype, as evidenced by the constant expression of α-SMA.

To investigate whether miR-9-5p and miR-221-3p can directly affect HSC activation and procollagen expression, we transfected LX-2 with miR-9-5p mimic and miR-221-3p mimic and examined the mRNA level of α-SMA and Col-1α1. As shown in Figure 6b, overexpression of miR-9-5p and miR-221-3p significantly downregulated the mRNA levels of both α-SMA and Col-1α1, suggesting that both miR-9-5p and miR-221-3p can directly inhibit HSC activation and decrease procollagen transcription, and thus the observed inactivation of HSCs (Figure 5) and alleviation of liver fibrosis (Figure 4) by hMSCs overexpressing miR-9-5p or miR-221-3p may at least be partially attributed to the direct action of miR-9-5p and miR-221-3p. Further investigations are required to delineate the direct targets of miR-9-5p and miR-221-3p and the downstream molecular pathways involved.

### 2.5. hMSCs Overexpressing miR-9-5p or miR-221-3p Decrease the Expression of Fibrogenic and Proinflammatory Factors in Injured Liver

**Figure 7:**
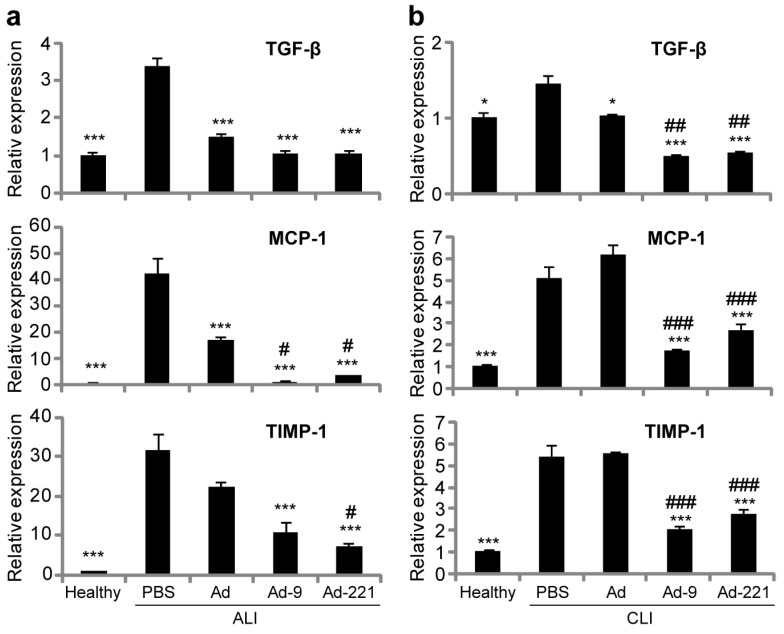
Transcriptional expression of fibrogenic and proinflammatory factors in the liver after infusion of hMSCs for 7 d. (**a**) In acutely injured livers (*n* = 5 per group). (**b**) In chronically injured livers (*n* = 5 per group). Expression was normalized to *GAPDH* and expressed as a fold change of Healthy. Data presented are the mean ± SEM from at least three independent experiments (* *p <* 0.05, *** *p <* 0.001, compared with PBS; # *p <* 0.05, ## *p <* 0.01, ### *p <* 0.001, compared with Ad).

Transforming growth factor β (TGF-β), tissue inhibitors of metalloproteinases 1 (TIMP-1), and monocyte chemotactic protein 1 (MCP-1), which function as either fibrogenic or proinflammatory factors that orchestrate HSC activation and participate in the progression of liver disease, were found to be significantly upregulated in the liver upon acute or chronic injury (Figure 7), as previously reported [29,31,32,33,34,35]. In animals with acute injury, administration of hMSCs decreased the expression of TGF-β and MCP-1, but not of TIMP-1, while hMSCs overexpressing miR-9-5p or miR-221-3p dramatically decreased the expression of all three factors (Figure 7a). In animals with chronic injury, hMSCs only decreased the expression of TGF-β, while again, hMSCs overexpressing miR-9-5p or miR-221-3p remarkably decreased all three factors (Figure 7b). These results suggest that the downregulation of TGF-β, MCP-1, and TIMP-1 contributes to the alleviation of liver injury via the overexpression of miR-9-5p or miR-221-3p in hMSCs.

## 3. Discussion

A variety of factors may lead to acute injury or even acute failure of the liver, and long-term repeated injury can lead to fibrosis, cirrhosis, and finally chronic liver failure. Liver failure is life-threatening and currently, the most effective treatment is liver transplantation, which is severely restricted because of a lack of donor organs and rapid progression of acute liver failure. MSCs have shown great potential as an alternative transplant for liver failure and cirrhosis. Both animal studies and short-term clinical trials have shown satisfactory outcomes such as improved survival rates, reduced severity scores, and improved liver synthetic and excretory functions, but long-term clinical therapeutic efficacy requires further elevation [36,37,38,39]. Sorting and/or priming of MSCs with growth factors or genetic modification of MSCs to improve their homing, survival, and proliferation ability resulted in better therapeutic efficacy [8,13,40]. In this study, we found that overexpression of miR-9-5p or miR-221-3p promoted hMSC homing to injured livers and improved their engraftment and therapeutic efficacy.

Studies have shown that pre-treatment of MSCs with HGF or overexpression of c-Met in MSCs promotes their migration and improves their repair of liver injury by increasing their homing, while inhibition of HGF/c-Met by c-Met inhibitor SU11274 decreases MSC migration and weakens their amelioration of liver injuries [21,22,23]. Our previous data showed that miR-9-5p and miR-221-3p promote the migration of rat bone marrow-derived MSCs toward HGF [25,26]. Consistently, in this study, we detected a higher migration ability of hMSCs overexpressing miR-9-5p or miR-221-3p toward HGF (Figure 1). Further in vivo study revealed that, after peripheral infusion in mice with acute or chronic liver injury, which have greatly elevated expression of HGF in the serum and the liver (Supplementary Figure S4), hMSCs overexpressing miR-9-5p or miR-221-3p resulted in higher engraftment than unmodified hMSCs (Figure 2), demonstrating that HGF recruits hMSCs to the injury sites, and that miR-9-5p and miR-221-3p promote HGF-induced hMSCs homing, which, in turn, resulted in higher engraftment of hMSCs, leading to more effective alleviation of liver injuries than unmodified hMSCs, as evidenced by the significantly lower liver/body weight ratio (Supplementary Figure S5), less hepatocyte necrosis and inflammatory infiltration (Figure 3), as well as, for chronic injury, the improvement in liver appearance and texture and the significant reduction in ECM deposition (Figure 4).

In our efforts to delineate the underlying mechanism, we found that the expression of α-SMA, which is upregulated upon liver injury and indicates the activation of HSCs, was further downregulated when receiving hMSCs overexpressing miR-9-5p or miR-221-3p (Figure 5), suggesting that inactivation of HSCs mediates the alleviation of liver injury by miR-9-5p- or miR-221-3p-modified hMSCs. Although in vitro treatment with CM from hMSCs overexpressing miR-9-5p or miR-221-3p failed to decrease the mRNA level of α-SMA and reverse the activated phenotype of human stellate cell line LX-2, the mRNA level of Col-1α1 was significantly downregulated compared to control CM (Figure 6a), indicating that hMSCs overexpressing miR-9-5p or miR-221-3p may alleviate liver fibrosis via their paracrine factors to disturb the expression of procollagen in activated HSCs. Moreover, upregulation of miR-9-5p or miR-221-3p in LX-2 significantly decreased the expression of α-SMA and Col-1α1 (Figure 6b), suggesting that direct action of miR-9-5p and miR-221-3p may partially mediate the improved therapeutic efficacy of hMSCs overexpressing miR-9-5p or miR-221-3p.

Upon liver injury, HSCs are activated and produce ECM to initiate a wound-healing response by encapsulating the injury. Activated HSCs release TGF-β and MCP-1, which serve as fibrogenic cytokine and chemoattractant, respectively, promote ECM production, recruit inflammatory cells, maintain inflammatory infiltration, and promote HSC chemotaxis and activation [32,33,41,42,43]. Activated HSCs stimulate the inflammatory response, and inflammation amplifies fibrosis, forming a positive feedback loop [44]. In this study, we found that infusion of hMSCs could decrease TGF-β and MCP-1 upon acute injury but could not decrease MCP-1 upon chronic injury. By contrast, overexpression of miR-9-5p or miR-221-3p resulted in a significant decrease in both factors upon injury (Figure 7), suggesting that downregulation of both TGF-β and MCP-1 may contribute to improved therapeutic effects by hMSCs overexpressing miR-9-5p or miR-221-3p.

In addition to promoting ECM synthesis, activated HSCs also secrete TIMP-1 to inhibit its degradation. Matrix metalloproteinases (MMPs) are among the key enzymes responsible for ECM degradation [43]. As a specific inhibitor of MMPs, upregulation of TIMP-1 inhibits ECM degradation and accelerates fibrosis progression [8,43]. In this study, TIMP-1 was significantly downregulated by hMSCs overexpressing miR-9-5p or miR-221-3p in both acutely and chronically injured animals, while its expression remained high when receiving unmodified hMSCs (Figure 7), suggesting that downregulation of TIMP-1 may greatly contribute to alleviating fibrosis and other hepatic injury by miR-9-5p- or miR-221-3p-modified hMSCs (Figure 3 and Figure 4).

Accumulating evidence suggests that MSCs can efficiently alleviate liver injuries through the secretion of paracrine factors, including noncoding microRNAs [8,45]. This led to an assumption that miR-9-5p and miR-221-3p may be transferred from hMSCs to adjacent liver cells and directly lead to the downregulation of fibrogenic and the proinflammatory factors TGF-β, MCP-1, and TIMP-1. Further investigations are required to detail the underlying molecular and cellular mechanisms, as none of these three factors are included in the putative mRNA targets of miR-9-5p and miR-221-3p predicted by TargetScan (https://www.targetscan.org (accessed on 27 May, 2024)) and miRDB (http://mirdb.org (accessed on 27 May 2024).

Previous studies have shown that miR-9-5p was downregulated in fibrotic liver tissues and activated HSCs and that overexpression of miR-9-5p inhibited the activation of HSCs and abrogated CCl_4_-induced hepatic fibrosis [46]. Consistently, in this study, transfection of LX-2 with miR-9-5p mimic significantly decreased the mRNA levels of both α-SMA and Col-1α1 (Figure 6b), suggesting that the alleviation of fibrosis by hMSCs overexpressing miR-9-5p may at least be partially attributed to miR-9-5p transfer from hMSCs to inhibit the activation of HSCs. Unlike miR-9-5p, miR-221-3p was reported to be upregulated in primary HSCs upon activation (6.1–26.8 folds) and expressed in a fibrosis progression-dependent manner in the human liver (1.8-fold increase) [47]; hepatocyte-specific suppression of miR-221-3p facilitated recovery from chronic injury and alleviated liver fibrosis [48]. In this study, we observed effective alleviation of liver fibrosis upon infusion of hMSCs overexpressing miR-221-3p (Figure 4), and upregulation of miR-221-3p in LX-2 (~45-fold) led to a significant decrease in both α-SMA and Col-1α1 mRNAs (Figure 6b), suggesting that upregulation of miR-221-3p may directly prevent HSC activation and procollagen expression, and its function in liver fibrosis may be cell type- and dose-dependent.

## 4. Materials and Methods

### 4.1. Isolation and Cultivation of hMSCs

Primary mesenchymal stem cells derived from Wharton’s jelly of neonatal human umbilical cords (hMSCs) were generously donated, with appropriate ethical approval, by the Stem Cell and Tissue Engineering Research Center, Guizhou Medical University, China. All experimental protocols were approved by the Ethics Committee of Guizhou Medical University and the Research Ethics Board of Soochow University and carried out in accordance with the Code of Ethics of the World Medical Association (Declaration of Helsinki). Fresh human umbilical cords were obtained after birth, after obtaining signed informed consent from all the donors. After removal of the outer layer and vessels, the reserved Wharton’s jelly of the umbilical cord was cut into 1 mm^3^ cubes and inoculated into a 75 cm^2^ culture flask (Corning, Durham, NC, USA) with standard culture medium composed of α-MEM (Gibco, Grand Island, NY, USA) supplemented with 10% heat-inactivated fetal bovine serum (FBS, Gibco, Auckland, Newzealand), 2 mM L-glutamine, 100 units/mL penicillin, and 100 μg/mL streptomycin (Gibco, Grand Island, NY, USA). The cells were incubated in a humidified 5% CO_2_ atmosphere at 37 °C, with the culture medium half replaced on the 3 d, and then completely replaced every 3 d thereafter. Tissue cubes were removed from the culture flasks upon 50% confluence of hMSCs. Once they reached 80–90% confluence, hMSCs were dissociated with a 0.25% trypsin–EDTA (Sigma-Aldrich, St. Louis, MO, USA) and subcultured in a 1:2 ratio. The expanded cells were positive for CD90, CD44, CD105, and CD73, but not for CD34 and CD45, and possessed osteogenic, chondrogenic, and adipogenic differentiation potential (Supplementary Figure S1). Cells in passages 4–10 were used for the experiments.

### 4.2. Upregulation of miR-9-5p or miR-221-3p in hMSCs

Upregulation of miR-9-5p or miR-221-3p in hMSCs was achieved via infection with recombinant adenovirus carrying the precursor sequence of miRNA and transfection with miRNA mimic. Construction and packaging of recombinant adenoviral vectors carrying the precursor sequences of miR-9 or miR-221 were performed as previously reported [25,26]. The recombinant adenoviruses expressing miR-9-5p (Ad-9), miR-221-3p (Ad-221), and the control virus (Ad), which has no inserted precursor sequence, were tittered as previously described [25], and the highest infection efficiency was approximately 80% at a multiplicity of infection (MOI) of 150. hMSCs at a density of 1 × 10^6^ cells per well were incubated with adenoviruses at an MOI of 150 for 1.5 h, replaced with the standard culture medium, and cultured as usual for another 48 h.

Transfection of miRNA mimic or the negative control (NC) was performed according to the manufacturer’s instructions (RiboBio, Guangzhou, China). Briefly, hMSCs were transfected with miR-9-5p mimic (50 nM), miR-221-3p mimic (50 nM), or NC (50 nM) using Lipofectamine 2000 transfection reagent (Invitrogen, Carlsbad, CA, USA) 24 h after plating. At 6 h post-transfection, the medium was replaced with a standard culture medium and the cells were incubated as usual for another 48 h.

Because the miRNA mimic we used lacked molecular markers, to track cell engraftment after transplantation, we incubated the cells with 10 μg/mL H33342 (bisBenzimide H 33342, Sigma-Aldrich, St. Louis, MO, USA) for 2 h, suspended them in cold PBS, and transplanted them at a dose of 1.0 × 10^7^ cells/kg body weight via tail vein injection. The remaining H33342-labeled cell suspensions were inoculated into new dishes and cultured with a standard medium to evaluate cell viability; the results showed that H33342 had no effect on the viability of cells.

### 4.3. Preparation of Conditioned Medium for hMSCs

For the preparation of conditioned medium (CM), hMSCs were transfected with miR-9-5p or miR-221-3p mimic for 48 h and reached 80–90% confluence. After washing with PBS, cells were maintained in α-MEM supplemented with 0.1% FBS and 2 mM glutamine for another 48 h. CM was collected and centrifuged at 2000× *g* for 20 min to remove cell debris. After being filtered through a 0.22 μm filter (Millipore, Carrigtwohill, Ireland), CM was used for subsequent experiments or stored at −80 °C.

### 4.4. Treatment of The Human Hepatic Stellate Cell Line LX-2

Human hepatic stellate cell line LX-2 was cultured in Dulbecco’s Modified Eagle Medium (DMEM; Gibco, Grand Island, NY, USA) supplemented with 10% FBS (Gibco, Auckland, Newzealand) and 2 mM L-glutamine at 37 °C in a humidified 5% CO_2_ atmosphere. To evaluate the effect of hMSC–CM on LX-2, LX-2 cells at 60–70% confluence were maintained in hMSC–CM for 48 h. To examine the effect of miR-9-5p or miR-221-3p on HSC activation and procollagen expression, LX-2 at 60–70% confluence was transfected with miR-9-5p mimic or miR-221-3p mimic at a final concentration of 50 nM using Lipofectamine 2000 transfection reagent. Forty-eight hours later, the transcription levels of α-SMA and Collagen-1α1 (Col-1α1) in LX-2 were quantified by qRT-PCR.

### 4.5. Analysis of Cell Proliferation

hMSCs (2000 cells/well) overexpressing miR-9-5p or miR-221-3p were seeded in a 96-well plate with α-MEM containing 10% FBS and incubated in a humidified 5% CO_2_ atmosphere at 37 °C. A 100 μL solution composed of 10 μL CCK-8 (Dojindo, Kumamoto, Japan) and 90 μL α-MEM was added to each well. After 2 h of incubation, absorbance at 450 nm and a reference at 650 nm was measured using a microplate reader (BioTek, Winooski, VT, USA). Measurements were performed in triplicate and repeated in three independent experiments.

### 4.6. Detection of Apoptosis and Death Using Flow Cytometry

Cell apoptosis and death were determined by double staining with Alexa Fluor 647-conjugated Annexin-V and propidium iodide (PI; Fcmacs, Nanjing, China). hMSCs were seeded at a density of 2 × 10^4^ cells/cm^2^ in a 6-well plate with α-MEM containing 10% FBS and processed according to the manufacturer’s instructions. Ten thousand cells per specimen were acquired using a FACS flow cytometer (FACScan), and cell fluorescence was analyzed using CellQuest Pro software 5.1(BD Biosciences, San Jose, CA, USA). The analysis was performed in triplicate in three independent experiments.

### 4.7. RNA Isolation and Quantitative Analysis Using Real-Time PCR (qPCR)

Total RNA was extracted using TRIzol^®^ Reagent (Ambion, Carlsbad, CA, USA) and treated with RQ1 RNase-free DNase (Promega, Madison, WI, USA) according to the manufacturer’s instructions. cDNA was generated with the RevertAid^TM^ First Strand cDNA Synthesis Kit (Takara, Kusatsu, Japan) using specific stem-loop RT primers for miRNAs (RiboBio, Guangzhou, China) and oligo(dT)_18_ primers (Thermo Fisher Scientific, Carlsbad, CA, USA) for mRNAs. Real-time PCR was performed in a Bio-Rad CFX96 system using SsoFast EvaGrean Supermix (Bio-Rad, Foster, CA, USA). Specific Bulge-Loop forward and reverse primers (RiboBio, Guangzhou, China) were used to amplify miR-9-5p, miR-221-3p, and snRNA U6 (snU6), which was used as an internal control for the normalization of miRNAs. Specific forward (F) and reverse^®^ primers for quantitative transcriptional analysis of HGF, transforming growth factor β (TGF-β), tissue inhibitor of metalloproteinase 1 (TIMP-1), and monocyte chemotactic protein 1 (MCP-1) were listed (Supplementary Table S1). *GAPDH* was used as an internal control for the normalization of mRNAs. Data were calculated in the Bio-Rad CFX manager software v2.1 using the ΔΔ*C*_T_ method (Livak and Schmittgen 2001) and expressed as relative quantities after snU6 or *GAPDH* normalization.

### 4.8. Migration Assays

Boyden Chamber. Transfilter migration of hMSCs was studied in a 48-well modified Boyden chamber (NeuroProbe, Gaithersburg, MD, USA). After a 30 min starvation in serum-free medium, hMSCs were digested with 0.25% trypsin–EDTA resuspended in α-DMEM and adjusted to 8 × 10^5^ cells/mL. A cell suspension of 50 μL was seeded in the upper chamber of each well on a poly-L-lysine (PLL; Sigma-Aldrich, St. Louis, MO, USA)-precoated polyvinylpyrrolidone-free polycarbonate membrane (12 μm pore size; Osmonics, Livermore, CA, USA), and 30 μL of α-MEM with or without 50 ng/mL HGF (PeproTech, Cranbury, NJ, USA) was placed in the lower chamber. The apparatus was incubated for 6 h at 37 °C in a 5% CO_2_ humidified incubator. After incubation, the chamber was disassembled, the upper side of the filter was wiped off, and cells attached to the lower side were fixed in 4% paraformaldehyde in 0.1 M PBS, pH 7.2, stained for 30 min in 0.1% cresyl violet (Solarbio, Beijing, China), and counted under 200× magnification in all fields of each well (each condition was run in six wells in each assay).

Dunn Chamber. The migration speed and efficiency of individual hMSCs were studied using a Dunn chamber (Hawksley, Sussex, UK), which is made from a Helber bacteria counting chamber by grinding a circular well in the central platform to leave a 1 mm wide annular bridge between the inner and outer wells. Once added to the outer well of the Dunn chamber, chemoattractants diffuse across the bridge to the inner well and form a steady concentration gradient that is stable for ~30 h thereafter. This apparatus allows for the direct monitoring of cell locomotion and the analysis of migration velocity, turning behavior, and directionality of migration. A 24 × 24 mm coverslip with hMSCs was inverted onto the chamber, and cell migration was recorded on the annular bridge every 5 min using a ×10 objective from a Leica DMI 6000 B microscope (Leica, Wetzlar, Germany) for a period of 6 h at 37 °C and 5% CO_2_. Migration traces of all sparsely distributed cells recorded on the annular bridge were analyzed. The forward migration index (FMI), which represents the efficiency of forward migration during the 6 h recording period, was calculated as the ratio of forward progress (net distance the cell progressed in the direction of the HGF source) to the total path length (total distance the cell traveled through the field). Migration speed was calculated from each time-lapse interval (5 min) recorded during the 6 h period.

### 4.9. CCl_4_-Induced Acute and Chronic Liver Injury Mouse Models

All animal experiments were conducted in accordance with Chinese laws and the guidelines approved by the Ethics Committee of Soochow University. Male Kunming mice (20 ± 2 g, *n* = 140) were purchased from the Experimental Animal Center of Soochow University of China. Acute liver injury was induced by subcutaneous injection of CCl_4_ (1:1 diluted with olive oil; Sigma-Aldrich, St. Louis, MO, USA) at a dose of 5 mL/kg body weight once. Chronic liver injury was induced by subcutaneous injection of CCl_4_ (1:1 diluted with olive oil) at a dose of 3 mL/kg body weight twice a week for the first six weeks and once a week for the subsequent four weeks. Transplantation of MSCs was performed 24 h after the last CCl_4_ administration at a dose of 1.0 × 10^7^ cells/kg body weight via tail vein injection. The control group received a tail vein injection of the same volume of PBS. At 7 d after cell transplantation, the mice were sacrificed and blood and liver specimens were collected and subjected to biochemical and histochemical analyses.

### 4.10. Histochemical Staining of Liver Sections

Livers were collected 7 d after cell transplantation. Partial livers were embedded immersed in optimal cutting temperature compound (OCT) and cryosectioned (12 μm thick) to analyze transplanted cell retention in livers. The remaining liver specimens were fixed with 4% paraformalin (Sigma-Aldrich, St. Louis, MO, USA) and embedded in paraffin (Sigma-Aldrich, Shanghai, China). Tissue sections (5 μm thick) were mounted on slides and hematoxylin–eosin (HE) staining was performed to analyze hepatocyte injury and inflammatory cell infiltration. Masson’s trichrome staining was used to analyze the extent of fibrosis. The expression of α-SMA was examined using immunofluorescence staining to study the activation of hepatic stellate cells.

### 4.11. Statistical Analysis

Data are presented as the mean ± SEM. Statistical analyses included Student’s *t* test or analysis of variance (ANOVA), with *p* < 0.05 considered statistically significant.

## 5. Conclusions

Upregulation of miR-9-5p or miR-221-3p in hMSCs promotes their migration toward HGF in vitro and their homing to the injured liver after peripheral infusion, leading to higher engraftment and more effective alleviation of both acute and chronic liver injuries, as evidenced by less centrilobular hepatocyte necrosis, inflammatory infiltration, and fibrosis. The improvement of therapeutic efficacy may be attributed to inactivation of HSCs and downregulation of fibrogenic and proinflammatory factors TGF-β, MCP-1, and TIMP-1 by hMSC paracrine factors, and overexpression of miR-9-5p and miR-221-3p may at least partially contribute to reversing HSC inactivation. These results demonstrate that modifying miRNA expression to elevate hMSC migration toward HGF, thereby enhancing their homing and engraftment, provides a promising strategy for more efficient treatment of liver injuries.

## Data Availability

All data generated or analyzed in this study are included in this published article and its supplementary files. The data that support the findings of this study are available from the corresponding author upon reasonable request.

## Supplementary Materials

The following supporting information can be downloaded at: https://www.mdpi.com/article/10.3390/ijms25137235/s1.
