# Supplementary material for: miR-9-5p and miR-221-3p Promote Human Mesenchymal Stem Cells to Alleviate Carbon Tetrachloride-Induced Liver Injury by Enhancing Human Mesenchymal Stem Cell Engraftment and Inhibiting Hepatic Stellate Cell Activation
Source: Int J Mol Sci. 2024 Jun 30;25(13):7235. doi: 10.3390/ijms25137235 (PMC11241704; doi:10.3390/ijms25137235)

Supplementary materials

Supplementary Table S1. Specific primers used for qPCR.

Gene	Forward primer	Reverse primer
HGF	5’-TGGTGTTTCACAAGCAATCCAGA-3’	5’-CCGTTGCAGGTCATGCATTC-3’
TGF-β	5’-GACCCTGCCCCTATATTTGGA-3’	5’-GCCCGGGTTGTGTTGGT-3’
TIMP-1	5’-CGAGACCACCTTATACCAGCG-3’	5’-GGCGTACCGGATATCTGCG-3’
MCP-1	5’-GCAGTTAACGCCCCACTCA-3’	5’-CAGCCTACTCATTGGGATCATCTT-3’
GAPDH	5’-CAATGTGTCCGTCGTGGATCT-3’	5’-GTCCTCAGTGTAGCCCAAGATG-3’

Supplementary Figure S1. Characterization of hMSCs. (a) The long spindle-shaped or fibroblast-like appearance of hMSCs (Scale bar=100 μm). (b) hMSCs differentiated into the osteogenic lineage by Alizarin Red staining (Scale bar=100 μm). (c) hMSCs differentiated into the chondrogenic lineage by Alcian blue staining (Scale bar=100 μm). (d) hMSCs differentiated into the adipogenic lineage by oil red O staining (Scale bar=50 μm).

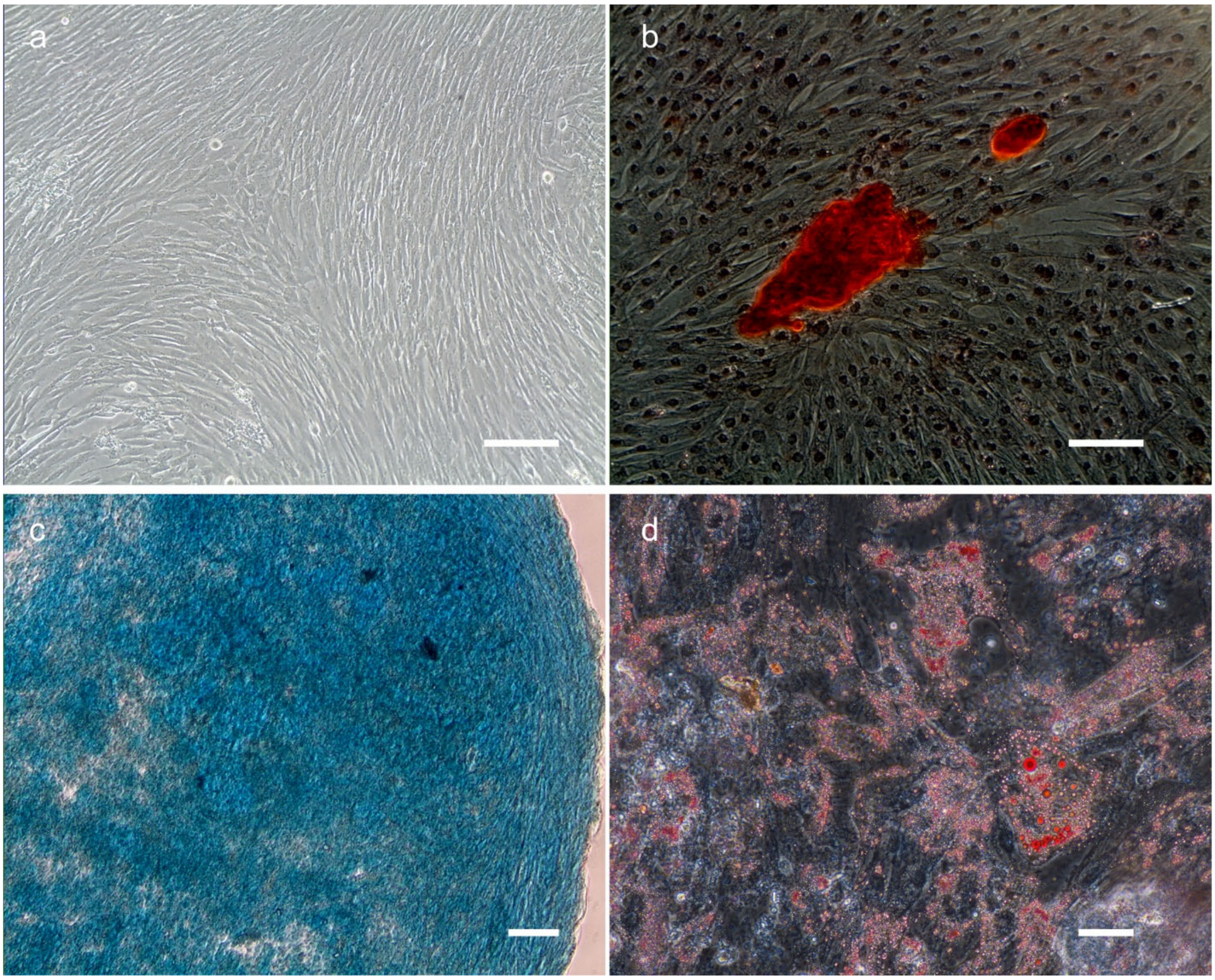

Supplementary Figure S2. Upregulation of miR-9-5p and miR-221-3p in hMSCs by adenovirus infection and miRNA mimic transfection. (**a**) Representative images of adenovirus (Ad) infected hMSCs (upper panel) and hMSCs transfected with miRNA mimic negative control (NC) and incubated with H33342 for 2 hours (lower panel). Scale bar = 250 μm. (**b**) Relative expression of miR-9-5p and miR-221-3p in hMSCs infected with Ad, Ad-9 and Ad-221, or transfected with NC, miR-9-5p mimic and miR-221-3p mimic, respectively. Data represent the mean ± SEM from at least three independent experiments (****p*<0.001, compared with Ad or NC).

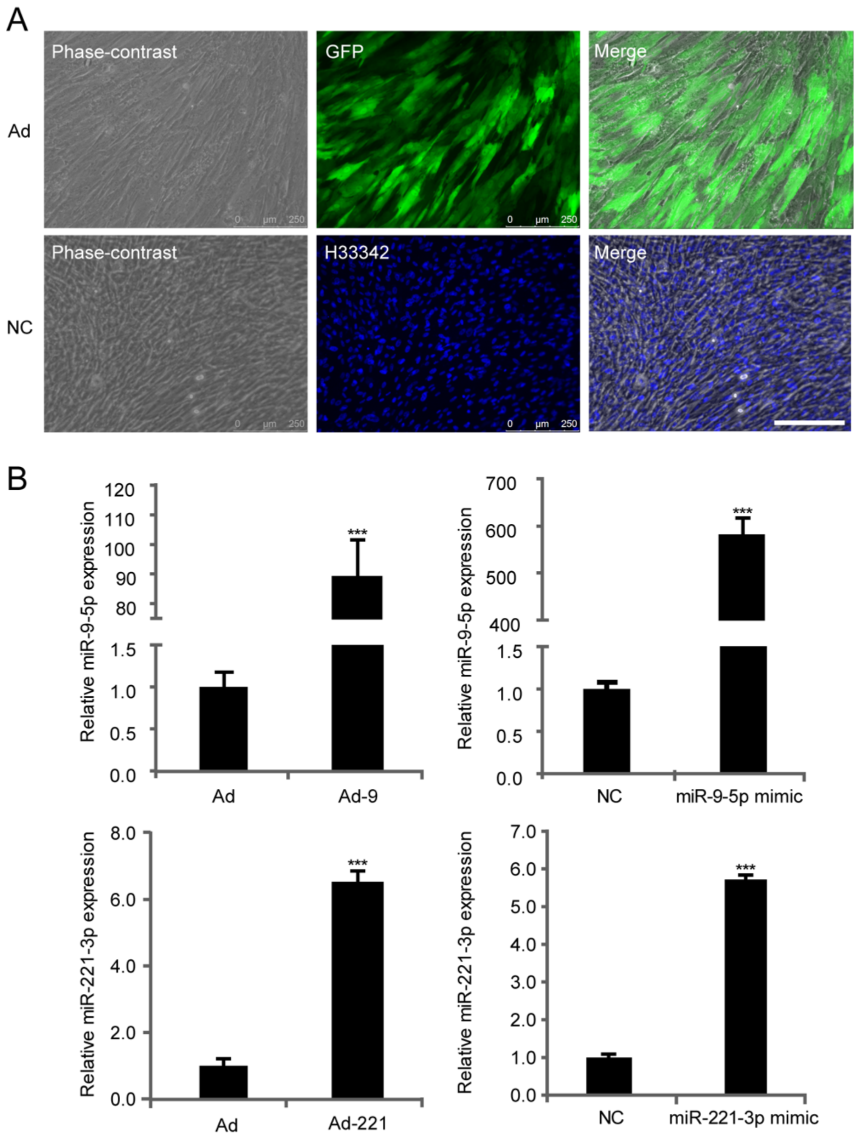

Supplementary Figure S3. Effects of miR-9-5p and miR-221-3p on the proliferation (a) and apoptosis (b) of hMSCs. (**a**) The OD value of hMSCs by CCK-8 proliferation assay. OD value = OD (450 nm) – OD (650 nm). (**b**) Cell apoptosis quantified by FACS analysis after staining with Annexin V and PI. The Annexin V^+ cells appeared in the apoptotic process. The percentage of apoptotic cells and the normalized cell viability (vs Ad or NC) in different groups were calculated. Data shown are mean ± SEM from at least three independent experiments.

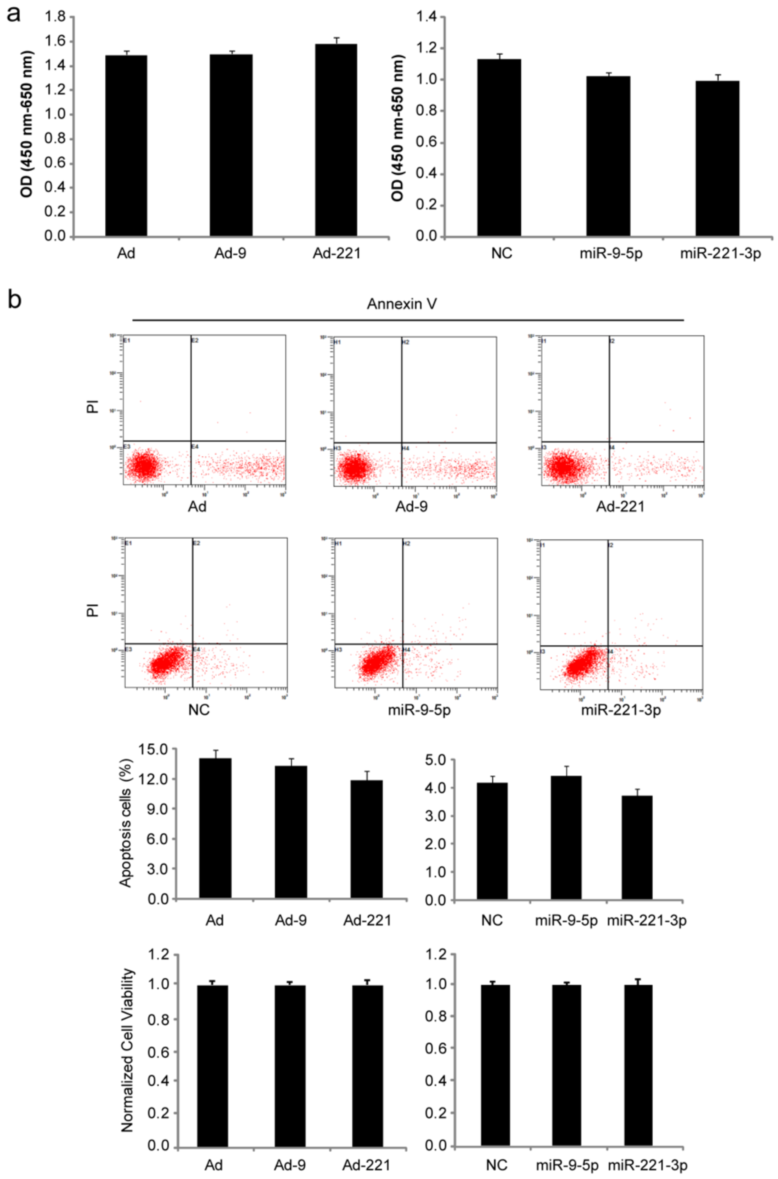

Supplementary Figure S4. Expression of HGF in mice with CCl_4-induced acute and chronic liver injuries (n = 5 per group). (**a**) Transcriptional expression of HGF in acute (ALI) and chronic (CLI) injured livers by RT-qPCR. (**b**) Protein level of HGF in serum from mice with acute and chronic liver injuries by ELISA. Data shown are mean ± SEM from at least three independent experiments (**p<0.01, *** p<0.001, compared with healthy).

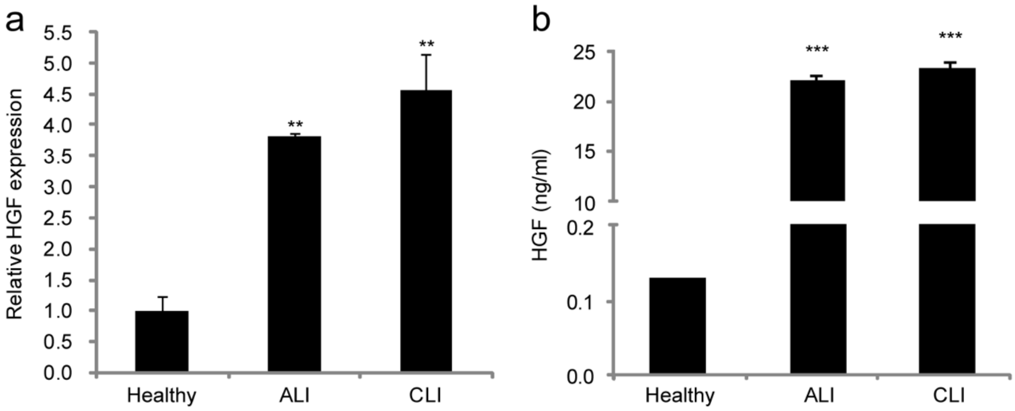

Supplementary Figure S5. Ratio of Liver/Body weight (%) in mice of healthy or with CCl_4-induced acute (**a**, **b**) or chronic (**c**, **d**) liver injury after cell transplantation for 7 d (n = 5 per group). For overexpression of miR-9-5p or miR-221-3p, hMSCs were infected with recombinant adenoviruses (**a**, **c**) or transfected with miRNA mimics (**b**, **d**). Control hMSCs were infected with Ad or transfected with NC. Data shown are mean ± SEM from three independent experiments (**p<0.01, *** p<0.001, compared with PBS. # p<0.05, ## p<0.01, compared with Ad or NC).

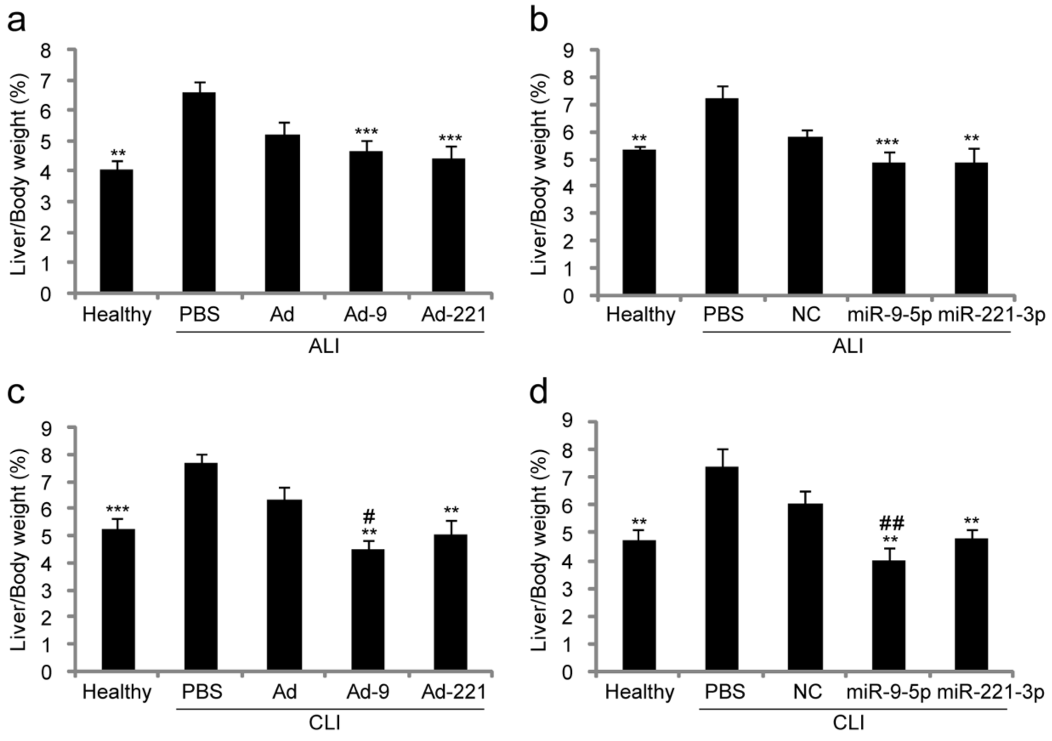

Supplement: Supplementary file 1 [file ijms-25-07235-s001.zip › Ijms-3055427-supplementary.pdf]
